# Beyond the Numbers: A Practical Framework for Interpreting the Full Blood Count for Foundation Doctors

**DOI:** 10.7759/cureus.117989

**Published:** 2026-10-15

**Authors:** Rosamma Lenitte Thomas

**Affiliations:** 1 Trauma and Orthopaedics, University Hospital Crosshouse, Glasgow, GBR

**Keywords:** clinical reasoning, foundation doctors, full blood count, haematological abnormalities, laboratory interpretation, medical education, pattern recognition

## Abstract

The full blood count (FBC) is one of the most frequently encountered investigations in clinical practice, yet interpreting an abnormal result requires more than recognising values outside a laboratory reference range. For foundation doctors and other early-career clinicians, the challenge is often determining whether an abnormality is clinically significant, whether it represents an acute or longstanding process, how abnormalities across different cell lines relate to one another, and when further investigation or escalation is required.

This narrative review presents the Beyond the Numbers framework, a practical approach to FBC interpretation that integrates laboratory findings with clinical urgency, previous results, patterns across cell lines and the wider clinical context. The framework begins with assessment of the patient's clinical condition. In an unwell or unstable patient, assessment, treatment or action, and escalation take priority, with investigation or verification undertaken in parallel where appropriate. In a clinically stable patient, interpretation proceeds through verification of the result, review of the trend, recognition of the pattern, consideration of the clinical context, and determination of the appropriate action.

The framework is applied to common red-cell, white-cell and platelet abnormalities and to situations in which multiple cell lines are affected. Particular emphasis is placed on recognising clinically important cross-line patterns, distinguishing potentially significant patterns from isolated numerical abnormalities, identifying when an FBC may not fit the clinical picture, and recognising situations that warrant senior or haematology input. The approach emphasises that patterns are prompts for further clinical reasoning rather than diagnostic equations, and that escalation does not require diagnostic certainty.

Beyond the Numbers provides a cognitive scaffold for moving from recognition of an abnormal FBC to proportionate clinical action. It is intended to complement, rather than replace, clinical judgement and disease-specific national or local guidance. Its potential value lies in making FBC interpretation more explicit, structured and teachable for clinicians developing practical reasoning skills.

Safe FBC interpretation requires integration of the number with the patient, the trajectory, the pattern and the clinical context. The Beyond the Numbers framework provides a practical educational approach to deciding when an FBC abnormality matters, what it may represent, and what should happen next. Prospective evaluation is required to determine its effect on interpretation, decision-making and appropriate escalation.

## Introduction and background

The full blood count (FBC) is one of the most frequently requested laboratory investigations in clinical practice and forms a fundamental component of patient assessment across medical and surgical specialties. It provides information on red cells, white cells, and platelets and may offer important clues to infection, inflammation, haematological disease, systemic illness, or acute clinical deterioration. For foundation doctors, interpreting FBC results is therefore a routine clinical responsibility and an important opportunity to recognise significant abnormalities, initiate appropriate investigation, and escalate care when required. However, the clinical value of the FBC lies not simply in identifying values outside the reference range, but in determining what those abnormalities mean for the individual patient in the context of their presentation, examination, previous results, and clinical trajectory [1,2].

Interpreting an abnormal FBC can nevertheless be challenging for early-career clinicians. Medical education provides a foundation in reference ranges and the pathophysiology of common haematological disorders, but translating this knowledge into real-time clinical decision-making is more complex. Clinicians must determine whether an abnormality is clinically significant, distinguish acute from chronic change, recognise patterns involving one or more cell lines, select appropriate initial investigations, and identify situations requiring urgent action or escalation. These decisions require clinical reasoning rather than isolated recognition of abnormal numbers. In particular, the significance of an abnormal FBC depends first on the clinical condition of the patient: an unwell patient may require immediate assessment, treatment, and escalation without delaying care to verify the laboratory result, whereas an unexpected abnormality in a clinically stable patient can be approached systematically through verification, review of previous results, pattern recognition, and interpretation in clinical context [1-4].

Existing reviews and educational resources provide general approaches to interpreting abnormal blood counts [1-3], while clinical guidelines from organisations such as the British Society for Haematology (BSH) and the National Institute for Health and Care Excellence (NICE) provide evidence-based recommendations for specific disorders and clinical scenarios [4,5]. Building on these resources, the Beyond the Numbers framework focuses specifically on the practical transition from identifying an abnormal FBC to deciding what should happen next. It places an explicit clinical-urgency gate before laboratory interpretation and integrates verification, trend assessment, pattern recognition, clinical context and escalation within a single bedside reasoning framework.

This educational narrative review applies the framework to common red-cell, white-cell, platelet and multiline abnormalities encountered in adult clinical practice. It is primarily situated within UK practice and foundation training, and therefore draws particularly on NICE and BSH guidance and UK clinical pathways. Although the underlying reasoning principles may be applicable more broadly, specific thresholds, investigation pathways and escalation processes should be interpreted according to relevant national and local guidance. The framework is intended to support early-career clinicians in moving from laboratory findings to proportionate clinical action rather than to replace condition-specific guidance, specialist assessment or clinical judgement.

This educational narrative review was informed by an iterative, topic-based literature search using PubMed and relevant medical journals. Search terms included “full blood count”, “complete blood count”, “FBC”, “CBC”, “red blood cells”, “white blood cells”, “platelets”, “transfusion”, “red blood cell transfusion”, “platelet transfusion” and “foundation doctors”, alongside terms relevant to individual cell-line abnormalities. Sources were selected according to their clinical relevance to FBC interpretation, investigation, management and escalation decisions in adult practice, with particular emphasis on current UK national guidance, including NICE and BSH recommendations. References were organised using Zotero, and key findings were recorded in a literature spreadsheet to support the development of individual sections. The aim was to provide a practical educational synthesis rather than an exhaustive or systematic review.

## Review

Beyond the numbers framework: a practical approach to interpreting the full blood count

Interpretation of the FBC extends beyond recognising values outside the laboratory reference range. Although automated analysers provide rapid measurements of multiple haematological parameters, the clinical significance of these results depends on the patient's condition, previous results, the pattern of abnormalities, and the wider clinical context [1,2]. Contemporary approaches to FBC interpretation emphasise systematic assessment and clinical reasoning rather than reliance on isolated numerical thresholds [1,2]. Building on these principles, this review proposes the Beyond the Numbers Framework, a practical approach designed to support foundation doctors and other early-career clinicians in translating abnormal FBC results into appropriate clinical decisions.

The Clinical-Urgency Gate

The first question is not “How abnormal is the result?” but “Is the patient clinically unwell?”

This distinction determines how the FBC should be approached. If the patient is clinically unwell or unstable, immediate clinical assessment, treatment, and escalation take priority. Investigation and verification of the FBC should occur in parallel where appropriate and should not delay time-critical management. This urgency-first principle is consistent with current guidance for the assessment and early management of potentially serious acute illness, including suspected sepsis [4]. Conversely, when the patient is clinically stable, an unexpected or abnormal result can be approached systematically by verifying the result, reviewing previous results, recognising the pattern, interpreting it in context, and deciding on the appropriate next action. Clinical assessment should therefore precede interpretation of the numerical abnormality in all cases. The indication for the FBC, presenting symptoms, observations, Early Warning Score, examination findings, comorbidities, and overall clinical trajectory should be considered alongside the laboratory result. At the same time, clinicians should remain alert to potentially misleading results, including those associated with sample problems, analyser flags, or other pre-analytical and analytical issues [1]. In a clinically stable patient, such findings may warrant verification before further investigation or management decisions are made.

The framework can therefore be understood as two linked pathways rather than a single rigid sequence: urgent clinical assessment and action for the unwell patient; structured verification and interpretation for the clinically stable patient.

For the Clinically Stable Patient: Verify the Result

An unexpected FBC abnormality should first be considered in relation to the possibility of laboratory or sampling error. Verification may include reviewing the report for analyser comments or flags, checking the clinical circumstances in which the sample was obtained, and repeating the FBC when appropriate [1].

Verification is particularly important when a result is unexpected, markedly discordant with previous findings, or inconsistent with the clinical picture. However, verification should not become a reason to delay treatment in a patient whose clinical condition indicates that urgent action is required [4].

Review the Trend

An isolated FBC represents a single point in time and should, where possible, be considered alongside previous results. Reviewing earlier investigations can help distinguish acute change from a longstanding abnormality, identify clinically important trends, and establish whether a finding has already been investigated or represents the patient's established baseline [1,2].

Previous admissions, outpatient correspondence, laboratory trends, and prior specialist assessments may provide important context. A stable abnormality may require a different approach from a newly developing or rapidly worsening abnormality. Trend therefore adds a temporal dimension to interpretation and may be more informative than a single result considered in isolation [1,2].

Recognise the Affected Cell Line and Pattern

Once the result has been verified and its trajectory considered, interpretation should focus on identifying the affected cell line or combination of cell lines. Abnormalities may primarily involve red cells, white cells, or platelets, or may affect more than one lineage.

The relationship between parameters is often more clinically informative than any individual value. Haemoglobin (Hb) concentration, red-cell indices, white-cell differential counts, platelet count, and, where available, peripheral blood film findings should therefore be considered together. Patterns such as isolated microcytic anaemia, macrocytic anaemia with thrombocytopenia, neutropenia, or pancytopenia can help narrow the differential diagnosis and guide appropriate initial investigation [1,2].

Recognition of patterns also helps clinicians identify situations in which a broader process should be considered. Abnormalities involving multiple cell lines, for example, may suggest systemic illness, medication effects, nutritional deficiency, infection, or bone marrow pathology and should not be approached as a series of unrelated numerical abnormalities [1,2].

Interpret in Clinical Context

Laboratory findings acquire meaning from the patient in whom they occur. The FBC should therefore be interpreted alongside the presenting complaint, history, examination, observations, comorbidities, medications, recent procedures or treatment, and other relevant investigations [1].

The same numerical abnormality may have very different implications depending on the clinical setting. A falling Hb after surgery, neutropenia in a patient receiving cytotoxic treatment, or thrombocytopenia in a patient with suspected sepsis requires a different clinical response from an isolated, longstanding abnormality in an otherwise well patient. Interpretation should therefore combine the laboratory pattern with the patient's clinical trajectory rather than relying on numerical thresholds alone [1,2,4].

Decide on the Appropriate Action

The final purpose of interpretation is to determine what should happen next. Depending on the clinical context, the appropriate action may be to monitor the abnormality, repeat the FBC, request targeted investigations, review a peripheral blood film, discuss the findings with a senior clinician, seek haematology advice, or initiate urgent treatment and escalation.

The urgency of action should reflect both the patient's clinical condition and the characteristics of the FBC abnormality. Abnormal laboratory values should not trigger automatic intervention simply because they fall outside a reference range, while apparently modest abnormalities should not be dismissed when they occur in an unwell patient or represent a significant change from baseline. The framework is therefore intended as a structured aid to clinical reasoning rather than a diagnostic algorithm or substitute for condition-specific guidance and local clinical pathways.

The Beyond the Numbers Framework can be summarised in Figure 1.

**Figure 1:**
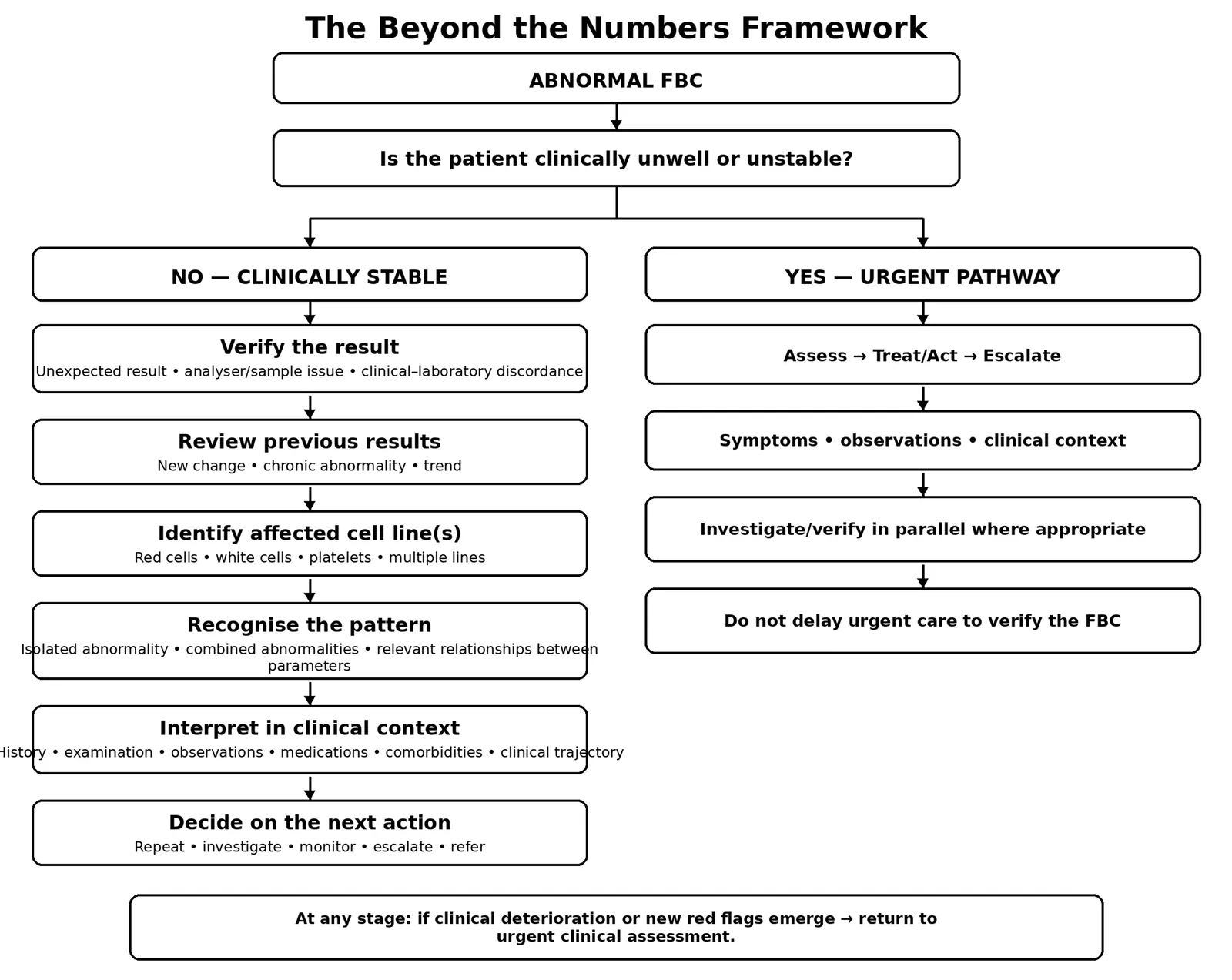
The Beyond the Numbers Framework Abbreviations: FBC, full blood count.

Red cell abnormalities

Haemoglobin and Anaemia: Why It Matters to Foundation Doctors

Hb is one of the most frequently reviewed components of the FBC and is central to the assessment of patients across medical and surgical specialties. For foundation doctors, Hb results are encountered routinely during daily patient reviews, peri-operative assessment, assessment of acute deterioration, and investigation of unexplained symptoms. Although Hb is a single numerical value, its interpretation may influence important clinical decisions, including further investigation, monitoring, escalation, and treatment [1-3,6].

The clinical importance of Hb interpretation lies not simply in identifying whether the value is outside the laboratory reference range, but in understanding what the abnormality represents in the individual patient. Anaemia is a manifestation of an underlying process rather than a diagnosis in itself. A reduced Hb concentration may result from acute or chronic blood loss, nutritional deficiency, inflammation, renal disease, haemolysis, bone marrow dysfunction, or other systemic disease processes. Safe interpretation therefore requires consideration of the patient's clinical condition, the trajectory of the Hb, the pattern of the red-cell indices, and the wider clinical context [1,2,6].

This distinction is particularly important in acute and surgical practice. A postoperative fall in Hb may represent expected blood loss, ongoing haemorrhage, haemodilution, or another pathological process, while the same Hb concentration in a clinically well patient with longstanding anaemia may require a very different response. Similarly, in medical practice, newly identified anaemia may be a clue to nutritional deficiency, chronic disease, renal disease, gastrointestinal blood loss, or other underlying pathology. For foundation doctors, the challenge is to distinguish these clinical presentations and identify the appropriate response [1,2,6].

The following sections apply the Beyond the Numbers Framework to red-cell abnormalities, with particular emphasis on anaemia, red-cell indices, iron deficiency, erythrocytosis, and transfusion.

Anaemia: Applying the Beyond the Numbers Framework in Clinical Practice

A practical application of the Beyond the Numbers framework to anaemia is summarised in Table 1.

**Table 1:** Beyond the Numbers: A Practical Approach to Anaemia Abbreviations: FBC, full blood count; Hb, haemoglobin; MCV, mean corpuscular volume; MCH, mean corpuscular haemoglobin; RDW, red cell distribution width.

Framework step	Key questions for the foundation doctor
Is the patient clinically unwell or unstable?	Is there evidence of active or significant bleeding? Is the patient symptomatic, for example with chest pain, dyspnoea, dizziness, or syncope? Are observations stable? Is there evidence of haemodynamic compromise? If yes: assess, treat, and escalate as clinically indicated; investigate and verify the FBC in parallel where appropriate. Do not delay urgent care simply to verify the result.
If clinically stable: verify the result	Is the result unexpected or inconsistent with the clinical picture? Could there be a sampling or laboratory issue? Does the result fit with the rest of the FBC and available clinical information?
Review the trend	What was the previous Hb level? Is this a new change, a gradual decline, or a longstanding abnormality? Has the anaemia been investigated previously? Are previous diagnoses, specialist reviews, or treatment plans documented?
Recognise the red-cell pattern	What are the MCV, MCH, and RDW? Does the pattern suggest microcytic, normocytic, or macrocytic anaemia? Is anaemia accompanied by abnormalities in other cell lines?
Interpret the pattern in context	What clinical conditions, medications, procedures, bleeding history, nutritional factors, or comorbidities could explain the findings? Does the pattern suggest a likely cause, or is further investigation required? Could there be more than one contributing process?
Decide the next action	Is repeat testing or monitoring appropriate? Are targeted investigations required, such as haematinics, renal function, inflammatory markers, blood film, or a haemolysis screen? Is senior or specialist advice required? If transfusion is being considered, what are the symptoms, clinical status, Hb trajectory, bleeding status, comorbidities, and relevant current guidelines?

Clinical pearl: A haemoglobin result is a clinical clue, not a diagnosis. Ask: Is the patient unwell? Is the result new? What pattern does the FBC show? What does the patient need next?

Red Cell Indices: Turning Numbers Into Patterns

The Hb concentration identifies anaemia, while the red-cell indices help describe its pattern. For foundation doctors, the most useful indices to recognise initially are the mean corpuscular volume (MCV), mean corpuscular haemoglobin (MCH), and red cell distribution width (RDW). These parameters can help direct the differential diagnosis when interpreted alongside the Hb concentration and clinical context [1-3,7,8].

The MCV describes the average size of red blood cells and provides a practical starting point for classifying anaemia as microcytic, normocytic, or macrocytic. The MCH reflects the average amount of Hb within an individual red cell and often follows the general pattern of the MCV. The RDW describes variation in red-cell size (anisocytosis) and may provide additional information when interpreted alongside the MCV [2,3,7,8].

For foundation doctors, these indices should be used as a pattern-recognition tool rather than a diagnosis. Common red-cell index patterns and their practical interpretation are summarised in Table 2.

**Table 2:** Quick reference: interpreting red-cell indices Abbreviations: FBC, full blood count; MCV, mean corpuscular volume; RDW, red cell distribution width.

FBC pattern	What it suggests	Common considerations	Useful next questions
Low MCV	Microcytic pattern	Iron deficiency, thalassaemia/haemoglobinopathy, some chronic inflammatory conditions	Is iron deficiency present? Is there evidence of blood loss? Are there relevant family or previous clinical findings?
Normal MCV	Normocytic pattern	Acute blood loss, chronic disease, renal disease, haemolysis, mixed causes	Is the anaemia new? Is there bleeding? Is renal function abnormal? Are other cell lines affected?
High MCV	Macrocytic pattern	Vitamin B12/folate deficiency, alcohol, liver disease, medications, marrow disorders	Are haematinic results available? Are medications or alcohol relevant? Are other FBC abnormalities present?
Raised RDW	Greater variation in red-cell size	Evolving nutritional deficiency or mixed red-cell populations	Does the RDW add information when considered with the MCV and clinical picture?

The MCV should therefore be considered a direction for investigation rather than an endpoint. A microcytic pattern may suggest iron deficiency but does not establish the diagnosis. Conversely, a normal MCV does not exclude iron deficiency, particularly when mixed causes of anaemia are present. Ferritin and, where appropriate, transferrin saturation provide additional information when assessing iron status [9,10]. In microcytic anaemia, the red blood cell count may provide an additional clue: a relatively preserved or elevated RBC count despite a low MCV can suggest thalassaemia trait, whereas a reduced RBC count may favour iron deficiency in the appropriate clinical context; neither finding is diagnostic in isolation [1,8].

Clinical pearl: Don't stop at the MCV. Hb ↓ → MCV → MCH/RDW → Clinical context → Targeted investigation. The practical question is: “What pattern does this FBC suggest, and what should I investigate next?”

Raised Haemoglobin and Erythrocytosis: When to Escalate

A raised Hb or haematocrit is encountered less frequently than anaemia in routine foundation practice, but an unexpected or persistent elevation should not simply be dismissed [1,6].

The initial approach is to consider whether the finding could represent relative erythrocytosis, for example because of reduced plasma volume, before considering causes of true erythrocytosis. Previous FBC results should be reviewed to establish whether the finding is new or persistent, and the clinical context should be considered, including smoking, respiratory disease, hypoxia, relevant medications, and other clinical features [1,6].

Useful initial questions include determining whether the finding is new or persistent, whether reduced plasma volume could explain the result, and whether there is evidence of hypoxia or underlying respiratory disease. The clinical assessment should also consider the presence of smoking, medication use, or other relevant risk factors, as well as whether other FBC parameters are abnormal. Finally, it is important to determine whether the elevation is persistent or remains unexplained.

Persistent or unexplained erythrocytosis should prompt appropriate senior review and, where indicated, specialist assessment. The purpose of this section is not to provide a detailed classification of erythrocytosis, but to ensure that foundation doctors recognise when a raised Hb requires further assessment [1,6].

Clinical pearl: A single raised Hb may be transient; a persistent unexplained elevation deserves an explanation.

Iron Deficiency Anaemia: A Common Diagnosis Not to Miss

Iron-deficiency anaemia (IDA) is an important practical diagnosis for foundation doctors because it is common across medical and surgical practice and may represent either a readily treatable deficiency or a marker of underlying disease. The key clinical principle is that identifying iron deficiency is not the same as identifying its cause [9,10].

A low Hb with a microcytic or hypochromic pattern may raise suspicion of iron deficiency, but the diagnosis should be supported by appropriate iron studies. Serum ferritin is an important marker of iron stores, although interpretation may be complicated by inflammation or chronic disease. Transferrin saturation can provide complementary information when ferritin interpretation is uncertain or inflammatory conditions are present [9,10].

Question 1: Is iron deficiency likely? The overall haematological pattern should be considered, including Hb, MCV, MCH, ferritin, transferrin saturation when appropriate, and relevant inflammatory markers in conjunction with the clinical context. A low ferritin strongly supports iron deficiency when interpreted within an appropriate clinical context. However, a normal or raised ferritin concentration does not necessarily exclude iron deficiency, particularly in the presence of inflammation or chronic disease, in which ferritin levels may be influenced by the underlying inflammatory state [9,10].

Question 2: Why is this patient iron deficient? Once iron deficiency has been identified, the next step is to determine why it has occurred. Potential contributing factors can be broadly categorised as blood loss, reduced iron intake or absorption, and increased iron requirements. Blood loss may arise from gastrointestinal bleeding, menstrual blood loss, postoperative bleeding, or other sources of ongoing blood loss. Reduced iron intake or absorption may result from poor nutritional intake, gastrointestinal disorders that impair iron absorption, or previous gastrointestinal surgery. Increased iron requirements may occur during pregnancy and in other clinical situations in which iron requirements are elevated, with assessment guided by relevant clinical guidance. A comprehensive assessment should also take into account the patient’s age, sex, symptoms, comorbidities, medications, and findings from previous investigations [9,10].

Question 3: Does the patient need further investigation? This is where “treating the number” can become unsafe. Identifying iron deficiency should prompt consideration of its underlying cause rather than treatment alone.

New IDA, particularly where gastrointestinal blood loss is possible, may require investigation for an underlying gastrointestinal cause. NICE guidance recommends offering quantitative faecal immunochemical testing (FIT) to adults with IDA to guide referral for suspected colorectal cancer, while ensuring that further assessment or referral is not delayed when clinical concern remains high [11].

Therefore, foundation doctors should not simply prescribe iron and close the episode without considering whether the underlying cause has been identified or whether further investigation and follow-up are required [9-11].

Question 4: How should iron deficiency be treated? For many patients, oral iron is the usual initial approach, provided it is appropriate and tolerated. Intravenous iron may be considered when oral iron is unsuitable, poorly tolerated, poorly absorbed, cannot be adhered to, or when there is insufficient time for oral therapy to be effective before surgery. Relevant guidance should be followed for the individual clinical setting [9,10].

Pregnancy and other specialist populations require consideration of their specific guidance rather than automatic application of a general adult pathway.

Clinical pearl: Iron deficiency is a finding, not the final diagnosis. Treat the deficiency, but also ask why it occurred.

For the foundation doctor, the practical sequence is: Recognise → Confirm → Find the cause → Treat → Follow up. The underlying cause may be considerably more clinically important than the anaemia itself [9,10].

Transfusion Principles: When Does the Number Matter?

A low Hb is one of the most clinically important abnormalities identified on an FBC, but Hb concentration alone does not determine whether a patient should receive a red-cell transfusion. Transfusion is therefore an important practical application of the Beyond the Numbers approach: the Hb result should be interpreted alongside the patient's clinical condition, symptoms, haemodynamic status, rate of change, cause of anaemia, active bleeding, comorbidities, and relevant transfusion guidance [12,13].

In a clinically stable adult without major haemorrhage or other circumstances requiring a different approach, current NICE guidance recommends a restrictive red-cell transfusion strategy, with a threshold around 70 g/L and a post-transfusion target of 70-90 g/L. A higher threshold may be appropriate in selected clinical circumstances, including acute coronary syndrome, according to current guidance. These thresholds should not be applied mechanically and do not replace clinical judgement or condition-specific transfusion pathways [12].

Key considerations for foundation doctors before red-cell transfusion are summarised in Table 3.

**Table 3:** Before Transfusing, Ask Abbreviations: FBC, full blood count; Hb, haemoglobin.

Question	What to consider
Is the patient clinically stable?	Symptoms attributable to anaemia, haemodynamic instability, chest pain, dyspnoea, or evidence of tissue hypoxia
Is there active bleeding?	Recent surgery, gastrointestinal bleeding, trauma, or another source of ongoing blood loss
What is the Hb trend?	Compare with previous FBCs: acute fall or chronic/stable anaemia?
What is causing the anaemia?	Blood loss, iron deficiency, chronic disease, haematinic deficiency, haemolysis, or another cause
Are important comorbidities present?	Particularly cardiovascular disease, heart failure, renal dysfunction, or factors affecting transfusion risk
What guidance applies?	Use current national and local transfusion guidance rather than relying on a memorised threshold
If transfusion is appropriate, what is the appropriate amount?	In a non-bleeding adult, a single-unit approach with subsequent clinical reassessment is generally recommended where appropriate

Transfusion addresses the immediate consequence of anaemia but does not necessarily treat its underlying cause. A patient with IDA, for example, may require investigation and iron replacement rather than repeated transfusion [9,10,12].

Safe transfusion also requires adherence to local transfusion policy, including appropriate patient identification, documentation, pre-transfusion checks, and bedside verification. Transfusion-associated circulatory overload (TACO) is an important preventable complication, particularly in patients with risk factors such as cardiac disease, renal dysfunction, fluid overload, or severe or chronic anaemia. Appropriate risk assessment, careful indication, and avoidance of unnecessary transfusion volume are therefore important components of safe practice [12,13].

Clinical Pearl: Do not transfuse the Hb. Assess the patient, understand why the Hb is low, decide whether transfusion is indicated, and then transfuse safely.

A practical sequence is: Low Hb → Assess clinical urgency → Review the trend → Identify the cause → Consider whether transfusion is indicated → Apply current guidance → Assess transfusion risk → Transfuse appropriately → Reassess.

Red-Cell Red Flags: When to Escalate

Early senior or specialist input should be considered when an FBC abnormality is associated with haemodynamic instability, suspected significant or ongoing bleeding, a rapidly falling Hb concentration, or severe or symptomatic anaemia. Specialist input is also appropriate when anaemia is accompanied by significant white-cell or platelet abnormalities, when there is uncertainty regarding the need for urgent transfusion, or when there is persistent or marked unexplained erythrocytosis. Further assessment should be considered when serious underlying pathology is suspected, including marrow disease or malignancy, or when significant cardiovascular or other comorbidity may affect interpretation or management. Early senior or specialist input is also warranted when an abnormality is inconsistent with the clinical picture and cannot readily be explained.

White-cell abnormalities: turning the differential into clinical context

White-cell abnormalities are among the most frequently encountered findings on the FBC and are particularly relevant to foundation doctors because they may provide an early laboratory signal of infection, inflammation, physiological stress, medication effects, or, less commonly, haematological disease. However, the total white-cell count (WBC) is a signal rather than a diagnosis. Its clinical value lies in identifying which cell population is responsible for the abnormality, whether the finding is new or persistent, and whether the laboratory pattern fits the patient's clinical presentation [1,2,6,14,15].

For foundation doctors, the practical question is therefore not simply “Is the WBC high or low?” but “Which white-cell population is abnormal, why might it be abnormal in this patient, and does it require action?” Both leukocytosis and leukopenia may occur in infection, while reactive and malignant processes can produce overlapping laboratory patterns [1,2,15].

Total White-Cell Count: The Starting Point

The total WBC provides an initial indication that further assessment may be required, but it should not be interpreted in isolation. Leukocytosis has a broad differential that includes infection, inflammation, physiological stress, surgery, trauma, medication effects, and haematological malignancy. Conversely, a normal or low WBC does not exclude serious infection [14,15].

The degree, duration and trend of the abnormality, together with the differential count and clinical context, help determine its significance. In acute practice, a raised WBC may accompany infection but should not automatically be equated with infection or sepsis. NICE guidance emphasises that WBC and other inflammatory markers should not be used as standalone diagnostic tests for sepsis; clinical assessment and physiological observations remain central [4].

A particularly useful question for foundation doctors is: “Is this an expected abnormality for the patient's current illness, or is there something about the result that does not fit?”

For example, a new neutrophilia in a clinically stable patient shortly after surgery may have a reactive explanation. In contrast, persistent or unexplained leukocytosis, marked abnormalities involving more than one cell line, constitutional symptoms, lymphadenopathy or splenomegaly should prompt further assessment [14,15].

Previous FBC results are particularly valuable. A longstanding abnormality with a documented explanation represents a different clinical problem from a previously normal WBC that has become substantially abnormal [1,14,15]. 

The Beyond the Numbers approach to an abnormal WBC is summarised in Table 4.

**Table 4:** Beyond the Numbers: a practical approach to an abnormal WBC Abbreviations: ANC, absolute neutrophil count; FBC, full blood count; WBC, white blood cell count.

Framework step	Key questions for the foundation doctor
Is the patient clinically unwell or deteriorating?	Are observations abnormal? Is there fever, hypotension, tachycardia, hypoxia, altered mental state or other evidence of deterioration? Is there a clinical concern for sepsis or another acute process? If yes: assess, treat and escalate as clinically indicated; investigate and verify the FBC in parallel where appropriate. Do not delay urgent care simply to verify the result.
If clinically stable: verify the result	Is the result unexpected or inconsistent with the clinical picture? Could there be a sampling or laboratory issue? Does the result fit with the rest of the FBC?
Review the trend	What was the previous WBC? Is this an acute change, a recurrent abnormality or a persistent finding? Is there a documented previous diagnosis or explanation?
Identify the cell population responsible	Are the neutrophils, lymphocytes, monocytes, eosinophils or basophils driving the abnormality? If neutropenic, what is the ANC?
Recognise the pattern and interpret the context	Does the pattern fit infection, inflammation, physiological stress, surgery, trauma, medication or another clinical explanation? Are there abnormalities in other cell lines? Are there constitutional symptoms, lymphadenopathy or splenomegaly?
Decide the next action	Is monitoring or repeat testing appropriate? Are further investigations or a blood film required? Does the patient require treatment of an underlying clinical problem? Is senior or haematology advice required? Does the patient meet criteria for an urgent sepsis or neutropenic-sepsis pathway?

Practical foundation-doctor message: Do not stop at “WBC high” or “WBC low”. Read the differential, assess the patient, compare the trend and ask whether the pattern has a plausible clinical explanation.

Neutrophils: Infection, Inflammation and Urgent Abnormalities

Neutrophils are often the most clinically informative component of the white-cell differential in acute hospital practice. Neutrophilia commonly accompanies infection and inflammation, but may also occur following surgery or trauma, during physiological stress, with corticosteroid treatment and in other reactive states. Neutropenia may result from infection, medication effects, viral illness, autoimmune disease, nutritional deficiency or disorders affecting neutrophil production or survival [15-17].

The total WBC, absolute neutrophil count (ANC), inflammatory markers, physiological observations, clinical examination and previous results should therefore be considered together. A neutrophil abnormality is a clinical signal rather than a diagnosis [15-17].

For neutrophilia, look for the explanation: When neutrophilia is identified, the appropriate first question is not “Which antibiotic should I prescribe?” but “Does the patient's clinical condition provide a plausible explanation for this result?”

Neutrophilia shortly after surgery may represent a reactive response, whereas the same finding in a patient with fever, hypotension or focal symptoms may warrant urgent assessment for infection. Timing, symptoms, observations, the remainder of the differential count and previous FBC results are therefore important [14,15].

The absence of neutrophilia also does not exclude serious infection, particularly in patients who are immunocompromised or neutropenic [4,15].

Persistent or marked neutrophilia without an adequate reactive explanation should prompt consideration of other causes, including medication effects and haematological disease. The finding becomes more concerning when persistent or unexplained, associated with abnormalities in other cell lines, or accompanied by constitutional symptoms, lymphadenopathy or splenomegaly [14,15].

When the number becomes urgent: Neutropenia requires particular attention because its clinical significance depends on its severity, duration and the condition of the patient. In adults, neutropenia is generally defined as an ANC below approximately 1.5 × 10⁹/L, with severe neutropenia generally below 0.5 × 10⁹/L; definitions and thresholds should be interpreted alongside the relevant clinical and laboratory guidance [16,17].

The foundation doctor should confirm the ANC rather than relying simply on the percentage of neutrophils. The assessment should consider whether the abnormality is acute or chronic and whether it is isolated or accompanied by other abnormalities. Common acquired causes include medications, viral infection, autoimmune disease, nutritional deficiency and disorders affecting neutrophil production or survival [16,17].

A clinically well patient with longstanding mild isolated neutropenia requires a different approach from a patient who has developed acute severe neutropenia while febrile or clinically deteriorating [16,17].

For neutropenia with suspected infection, treat as an emergency: The most important safety message for foundation doctors is the combination of neutropenia and suspected infection.

In patients receiving anticancer treatment, NICE guidance defines neutropenic sepsis using severe neutropenia together with fever or other signs and symptoms consistent with clinically significant sepsis, and recommends treating suspected neutropenic sepsis as an acute medical emergency [5].

The broader clinical principle is more important than memorising a definition: A clinically unwell neutropenic patient requires urgent assessment and escalation; do not wait for the neutrophil count to recover or for the source of infection to become obvious.

Initial assessment should include appropriate clinical examination and investigations, which may include FBC, renal and liver function, CRP, lactate and blood cultures according to the clinical situation. Empiric antimicrobial treatment should follow the current local antimicrobial and neutropenic-sepsis guideline, taking account of patient-specific and local microbiological factors [5].

This is consistent with the wider NICE approach to suspected sepsis: patients at high risk of severe illness or death require urgent assessment and, when indicated, prompt intravenous antibiotics [4,5]. Key questions when interpreting an abnormal neutrophil count are summarised in Table 5.

**Table 5:** Beyond the Numbers: interpreting an abnormal neutrophil count Abbreviations: ANC, absolute neutrophil count; WBC, white blood cell count.

Question	What the foundation doctor should consider
Is the patient clinically unwell?	Fever, hypotension, tachycardia, hypoxia, altered mental state or other evidence of deterioration
Is the neutrophil count high or low?	Identify neutrophilia versus neutropenia and check the ANC
Does the WBC support the pattern?	Compare the total WBC with neutrophils and the remainder of the differential
Is there an obvious reactive explanation?	Infection, inflammation, surgery, trauma, physiological stress or medication
What was the previous result?	Acute change, persistent abnormality or longstanding finding
If neutropenic, how severe is it?	Consider the ANC, duration and clinical context
Are there red flags?	Neutropenia with fever or clinical deterioration, persistent unexplained neutropenia, marked unexplained neutrophilia, other cytopenias, constitutional symptoms or organomegaly
What action is required?	Treat the clinical problem, investigate the source, repeat or monitor, seek senior advice, or activate the relevant sepsis/neutropenic-sepsis pathway

Clinical pearl: Neutrophilia asks: “What is driving this response?” Neutropenia asks: “How low is it, why is it low, and is the patient unwell?”

Lymphocytes: Reactive Versus Persistent

Lymphocyte abnormalities are best interpreted using the absolute lymphocyte count, clinical context and trend rather than treating a single abnormal result as a diagnosis [18-20].

Acute lymphocytosis is commonly reactive, particularly in the setting of infection, whereas persistent or unexplained lymphocytosis requires review of previous FBC results and consideration of chronic infection, medication effects and lymphoproliferative disorders [18-20]. For foundation doctors, the most useful distinction is between acute, persistent and clinically concerning abnormalities.

Acute lymphocytosis: Consider whether there is an acute infective or inflammatory illness, particularly a viral illness [18,20].

Persistent lymphocytosis: Review previous FBCs and determine whether the abnormality is persistent or increasing. Consider associated lymphadenopathy, splenomegaly or unexplained systemic symptoms [18-20].

Lymphopenia: A low lymphocyte count is often nonspecific and may occur during acute illness or in association with infection, inflammation, medications, immunosuppression and systemic disease. It should therefore be interpreted in context rather than automatically attributed to a specific diagnosis [21,22].

Lymphocyte abnormalities with other FBC changes: Anaemia, thrombocytopenia, abnormal neutrophils or other abnormalities may indicate a broader haematological process and should increase the threshold for senior or specialist review [15,19].

NICE guidance on suspected cancer should also be considered where persistent or unexplained lymphocyte abnormalities occur alongside features such as lymphadenopathy, hepatosplenomegaly, unexplained fever, recurrent infection, bruising or bleeding [11].

Clinical pearl: Do not diagnose from the lymphocyte count alone. Ask: acute or persistent? What was the previous count? What does the patient look like? Are there other abnormal cell lines or clinical features suggesting haematological disease?

Monocytes: Reactive Versus Persistent

Monocytosis has a broad differential and may occur with infection, inflammation and other reactive conditions, as well as haematological disease. For foundation doctors, the most useful distinction is transient/reactive versus persistent and unexplained monocytosis [23]. Consider the following points.

Transient or reactive monocytosis: Is there an acute infection, inflammatory condition or recent physiological stress that provides an explanation?

Persistent monocytosis: What do previous FBC results show? Is the abnormality sustained?

Persistent and unexplained monocytosis: Are there accompanying abnormalities such as anaemia, thrombocytopenia or abnormal neutrophils?

Concerning clinical features: Is there splenomegaly, constitutional symptoms or another reason to suspect an underlying haematological disorder?

Persistent monocytosis does not mean that a patient has a haematological malignancy. However, sustained unexplained monocytosis may be a presenting feature of disorders such as chronic myelomonocytic leukaemia (CMML) and may warrant further assessment, including blood-film review or haematology discussion according to local pathways [23].

Clinical pearl: For monocytes, persistence matters more than an isolated result. Review the trend, look at the rest of the FBC and ask whether the clinical picture provides a reactive explanation.

Eosinophils: Allergy, Infection and Persistent Elevation

Eosinophilia has a broad differential and is most commonly reactive. Important secondary causes include allergic disease, medication reactions, parasitic infection and inflammatory or autoimmune conditions. Less commonly, persistent eosinophilia may reflect a clonal haematological disorder [14,15].

For foundation doctors, the practical approach is to determine whether the abnormality is persistent and whether there is an appropriate clinical explanation. The assessment should consider the presence of allergic disease or asthma, any new medications or possible drug reactions, and relevant travel or exposure history that may suggest parasitic infection. Gastrointestinal, respiratory, dermatological, or other systemic symptoms should also be assessed, together with any evidence of inflammatory or autoimmune disease. Particular attention should be given to abnormalities that persist or remain markedly elevated despite the absence of an obvious secondary cause.

A history of atopy, medication exposure and travel may therefore be particularly informative. Persistent or marked eosinophilia deserves greater attention because eosinophil-mediated organ damage can occur and specialist assessment may be required. Current classifications generally regard an eosinophil count above 1.5 × 10⁹/L as hypereosinophilia, but numerical thresholds should be interpreted alongside the clinical picture and current guidance [14,15].

Clinical pearl: An eosinophil count is a clue, not a diagnosis. Ask what is driving it, whether it is persistent and whether there is evidence of organ involvement.

Basophils: Confirm, Contextualise and Escalate When Persistent

Basophils are the least numerous circulating white-cell population and are encountered less frequently in routine FBC interpretation than neutrophils or lymphocytes. An isolated abnormal basophil count is often of limited immediate clinical significance; however, persistent or unexplained basophilia can provide an important clue to underlying myeloid disease, particularly when accompanied by other abnormalities in the FBC [14,24].

For foundation doctors, the key is not to memorise rare causes but to recognise when the finding warrants further attention.

Is the abnormality genuine?: Review the absolute basophil count, laboratory reference range and previous FBC results. An unexpected result should be interpreted cautiously and, where appropriate, confirmed [15,24].

Is there a clinical or reactive explanation?: Consider allergic or inflammatory conditions and other potential reactive causes. The remainder of the differential count, symptoms and other FBC abnormalities may provide important clues [24].

Is it persistent or unexplained?: Persistent basophilia, particularly when accompanied by marked leukocytosis, left-shifted granulopoiesis, thrombocytosis, anaemia or other abnormalities, should prompt consideration of an underlying myeloid neoplasm and appropriate haematological assessment. Basophilia is particularly associated with chronic myeloid leukaemia and other myeloproliferative neoplasms [14,24].

Clinical pearl: Basophilia is rarely an isolated emergency. Persistence is the signal. For a single unexpected result, confirm and review. For persistent or unexplained basophilia, review the whole FBC and clinical context. For basophilia with other haematological abnormalities, consider myeloid disease and seek appropriate advice.

Practical considerations for abnormalities in the remaining white-cell differential are summarised in Table 6.

**Table 6:** Beyond the Numbers: What to Ask About Differential White-Cell Abnormalities Abbreviations: FBC, full blood count.

Finding	Questions to ask
Lymphocytosis	Acute or persistent? Is there a plausible reactive/infectious explanation? What do previous FBCs show? Any lymphadenopathy, splenomegaly or systemic symptoms?
Lymphopenia	Is this occurring in acute illness, infection, inflammation, medication/immunosuppression or systemic disease? Is it persistent? Are other cell lines affected?
Monocytosis	Transient/reactive or persistent? Is there an obvious inflammatory/infectious explanation? Is it unexplained or accompanied by anaemia, thrombocytopenia, abnormal neutrophils or splenomegaly?
Eosinophilia	Is it persistent? Atopy/asthma? New medication? Travel or exposure history? Systemic symptoms or possible organ involvement?
Basophilia	Is it genuine and persistent? Is there a reactive explanation? Are there other abnormalities suggesting a myeloid disorder?

WBC Red Flags: When an Abnormal Differential Needs Escalation

Neutropenia accompanied by fever or clinical deterioration requires urgent assessment for suspected neutropenic sepsis. Marked or rapidly changing leukocytosis, persistent unexplained white-cell abnormalities, circulating blasts or other concerning blood-film findings, and abnormalities accompanied by constitutional symptoms, lymphadenopathy or splenomegaly should prompt further assessment and consideration of senior or haematology advice. The degree of urgency depends on the patient's clinical condition and the nature of the abnormality.

Platelet abnormalities: from platelet count to bleeding and thrombosis risk

Platelets are central to primary haemostasis, and abnormalities in platelet number may present with bleeding, thrombosis, or as an incidental finding on routine blood testing. For foundation doctors, however, the platelet count should be viewed as a clinical signal rather than a diagnosis. Its significance depends on the degree of abnormality, the trend over time, the wider FBC, and, most importantly, the clinical condition of the patient [1,25,26].

Platelet Count: Is the Result Genuine and What Is the Pattern?

In most adult laboratories, the usual platelet reference range is approximately 150-450 × 10⁹/L, although the laboratory-specific reference interval should be used. An abnormal result should prompt interpretation rather than an automatic diagnosis or intervention [1,25,26].

For a clinically stable patient with an unexpected result, the first consideration is whether the platelet count is genuine. The laboratory report should be reviewed for analyser comments or flags, the clinical circumstances in which the sample was obtained should be considered, and previous results should be checked. Repeat testing or examination of a blood film may be appropriate when the result is unexpected or inconsistent with the clinical picture. Analytical and pre-analytical factors can occasionally produce misleading platelet counts; pseudothrombocytopenia, for example, may result from platelet aggregation in the sample [1,25].

The previous FBC also provides an important temporal dimension. A mildly abnormal but stable platelet count may have a very different significance from a rapidly falling count in a previously normal patient. Previous admissions, outpatient results and established baseline values should therefore be reviewed whenever available [1,25].

The platelet count should then be interpreted alongside the rest of the FBC. Concurrent anaemia, abnormalities of the WBC, or changes affecting more than one cell line may provide important clues to the underlying process. Isolated thrombocytopenia, thrombocytopenia with anaemia, and thrombocytosis accompanied by microcytosis, for example, prompt different clinical considerations [1,6,25].

Finally, the numerical abnormality must be interpreted in the patient. Is the patient bleeding or thrombosing? Are they acutely unwell, septic or post-operative? Is an invasive procedure planned? Have there been recent medication changes, transfusion or heparin exposure? These factors determine whether the appropriate response is observation, repeat testing, targeted investigation, treatment or escalation.

Clinical pearl: The platelet count is a starting point, not a diagnosis. Its meaning comes from the result, the trend, the wider FBC and the patient in whom it occurs.

Platelet Abnormalities: Applying Beyond the Numbers

The application of the Beyond the Numbers framework to platelet abnormalities is summarised in Table 7.

**Table 7:** Beyond the Numbers: A Practical Approach to Platelet Abnormalities FBC, full blood count

Framework step	Key questions for the foundation doctor
Assess clinical urgency	Is the patient clinically unwell, unstable or actively bleeding? Is there evidence of thrombosis or clinical deterioration? If the patient is unstable, assess, treat and escalate as clinically indicated. Investigate and verify the FBC in parallel where appropriate; do not delay urgent care to confirm the platelet count.
Verify the result	Is the platelet count genuine? Are there analyser flags, sample issues, platelet clumping or a discrepancy between the laboratory result and the clinical picture?
Review the trend	Is the platelet abnormality new, longstanding, stable or changing rapidly? What do previous FBC results show?
Recognise the pattern	Is the platelet abnormality isolated, or are haemoglobin, white-cell counts or blood-film findings also abnormal?
Interpret in clinical context	Is there bleeding, thrombosis, infection, inflammation, recent surgery, medication exposure, heparin exposure or a planned invasive procedure?
Decide the next action	Should the FBC be repeated, investigated further or monitored? Is treatment required? Does the patient need senior or haematology advice or urgent escalation?

Thrombocytopenia: Is the Patient at Risk of Bleeding, and Why Is the Count Low?

Thrombocytopenia is usually defined as a platelet count below 150 × 10⁹/L. The important clinical question, however, is not simply whether the count is below the reference range, but whether the patient is at risk of clinically significant bleeding and why the platelet count is low. The risk of bleeding does not increase in a simple linear relationship with the platelet count; the clinical picture, rate of change, coexisting coagulation abnormalities, medications and underlying disease all influence risk [1,25,26].

In an unwell or bleeding patient, clinical assessment takes priority over verification of the numerical result. Features of bleeding include petechiae, purpura, mucosal bleeding, haematuria, gastrointestinal bleeding and heavy vaginal bleeding. More serious features include significant gastrointestinal or intracranial bleeding, haemodynamic instability or rapid clinical deterioration. Severe thrombocytopenia, particularly when accompanied by active bleeding, requires urgent assessment [1,26].

The degree of thrombocytopenia provides useful context but should not be used as an isolated predictor of bleeding. Patients with platelet counts above approximately 50 × 10⁹/L are often asymptomatic, whereas spontaneous serious bleeding becomes increasingly concerning at very low platelet counts. A platelet count below 10 × 10⁹/L is generally considered a high-risk finding and should prompt urgent clinical assessment. These thresholds should not replace clinical judgement, particularly in patients with additional haemostatic abnormalities or active bleeding [1,26].

Once immediate clinical concerns have been addressed, the cause of thrombocytopenia should be considered. Broad mechanisms include reduced platelet production, increased destruction or consumption, splenic sequestration, dilution and laboratory artefact. Common clinical contexts encountered by foundation doctors include acute infection or sepsis, liver disease and hypersplenism, alcohol use, medications, immune thrombocytopenia (ITP), malignancy or marrow disease, and dilution following major fluid or blood replacement [1,25,26].

The rest of the FBC and the blood film may substantially narrow the differential diagnosis. Isolated thrombocytopenia may suggest a different process from thrombocytopenia accompanied by anaemia, leukopenia, leukocytosis or abnormal cell morphology. A blood film is particularly important when platelet clumping is suspected or when there is concern about red-cell fragmentation. The presence of schistocytes in a patient with thrombocytopenia and evidence of haemolysis should raise concern for a thrombotic microangiopathy (TMA) and requires urgent senior assessment [1,6,27].

The trajectory is equally important. A new fall from a previously normal platelet count is more concerning than a stable, longstanding mild thrombocytopenia. The clinician should therefore review previous FBCs and ask about recent infection, surgery, hospitalisation, medication exposure and heparin exposure, as well as clinical features suggesting bleeding, thrombosis or systemic disease [1,28].

Particular patterns should prompt urgent consideration of alternative diagnoses. Thrombocytopenia accompanied by neurological or renal dysfunction, haemolysis or schistocytes, abnormal coagulation results, major bleeding, or recent heparin exposure may represent a haematological or thrombotic emergency. Importantly, heparin-induced thrombocytopenia (HIT) is a pro-thrombotic disorder, so thrombocytopenia after heparin exposure should not simply be interpreted as an increased bleeding risk [28].

For foundation doctors, the practical objective is therefore not to memorise every cause of thrombocytopenia, but to recognise the patient who is bleeding, the patient whose platelet count is changing rapidly, and the patient whose pattern suggests a potentially serious underlying process.

Thrombocytopenia: What Should I Ask?

When thrombocytopenia is identified, six practical questions can guide the initial bedside assessment. Is the patient bleeding, particularly with mucosal bleeding, petechiae, purpura or significant haemorrhage? How low is the platelet count, and is it falling rapidly or longstanding and stable? Is thrombocytopenia isolated, or are other cell lines or the blood film also abnormal? Could infection, medication or heparin exposure, liver or splenic disease, an immune process, dilution, consumption or marrow disease explain the finding [1,28]? Are there red flags such as haemolysis, schistocytes, renal or neurological dysfunction, abnormal coagulation, suspected HIT or clinical deterioration [6]? Does the patient require urgent senior or haematology assessment? In patients with thrombocytopenia who are receiving or being considered for prophylactic or therapeutic anticoagulation, the platelet count should prompt consideration of whether anticoagulation remains appropriate, taking account of bleeding and thrombotic risks and seeking senior or specialist advice in accordance with local guidance.

Clinical pearl: The platelet count tells you how many platelets are present; the bleeding phenotype, trend and clinical context tell you how worried you should be.

Falling Platelet Count: When Does the Trajectory Matter?

A falling platelet count can be clinically more informative than a single low value. A patient whose platelet count has remained mildly reduced for months is different from one whose count has fallen substantially over several days. The key questions are how much has the count fallen, over what period, and what has happened to the patient during that time?

Even when the platelet count remains within the laboratory reference range, a substantial decline from the patient's previous baseline may warrant attention. A precipitous fall should prompt review of the recent clinical course, medications, procedures and potential causes of platelet consumption or destruction [1,25].

In hospitalised patients, falling platelet counts commonly occur in the context of acute illness, infection or sepsis, major surgery, bleeding, dilution, liver disease or medication exposure. The pattern should therefore be interpreted alongside the overall clinical trajectory rather than attributed automatically to one cause. The timing of the fall in relation to new medications, procedures or heparin exposure is particularly important [1,25,28].

HIT is a key diagnosis not to miss. It is an immune-mediated, highly pro-thrombotic complication of heparin exposure. The important teaching point for foundation doctors is that HIT should be considered from the pattern and timing of the platelet fall together with thrombosis risk, rather than waiting for an extremely low platelet count. A patient with suspected HIT requires prompt senior assessment and management according to the appropriate diagnostic pathway [28].

Similarly, thrombocytopenia associated with haemolysis, schistocytes, new renal or neurological dysfunction, or other evidence of microangiopathy should prompt urgent assessment for a TMA. Thrombocytopenia occurring with abnormal coagulation and clinical deterioration may also indicate disseminated intravascular coagulation (DIC), particularly in severe infection or other critical illness [6,27,29].

Medication history is another essential component of interpreting a falling platelet count. New or recently changed medications should be reviewed, particularly when the fall is otherwise unexplained. Drug-induced thrombocytopenia can occur through several mechanisms, and recognising the temporal relationship between drug exposure and platelet decline may be more informative than the absolute count alone [1].

The trajectory should also influence how quickly the patient is reassessed. A stable mild abnormality without red flags may allow planned repeat testing and outpatient investigation, whereas a rapidly declining platelet count, particularly in a hospitalised or clinically deteriorating patient, warrants more urgent assessment. Patients with moderate-to-severe thrombocytopenia of unclear cause or a continuing decline may require haematology referral.

A falling platelet count does not, however, automatically indicate platelet transfusion. The decision depends on the clinical circumstances, including active bleeding, the severity and cause of thrombocytopenia, and whether an invasive procedure is planned. NICE advises that platelet transfusion decisions should take account of the clinical situation, including whether the platelet count is falling and whether there are other causes of abnormal haemostasis [12].

Thrombocytosis: Reactive or Persistent and Unexplained?

Thrombocytosis is generally defined as a platelet count above 450 × 10⁹/L. Many cases encountered in general clinical practice are secondary (reactive) rather than due to a primary bone-marrow disorder. Common reactive settings include acute or chronic infection and inflammation, recent surgery or tissue injury, blood loss, iron deficiency, malignancy and post-splenectomy states [1,30,31].

The key question is therefore why the platelet count is high, rather than whether the numerical abnormality itself requires treatment. A raised platelet count shortly after surgery, trauma or fracture, for example, may reflect a reactive response to tissue injury or inflammation. Timing and clinical context are important [30,31].

Iron deficiency is an especially useful cause to consider because it is common, potentially treatable, and may coexist with other abnormalities on the FBC. A raised platelet count accompanied by microcytosis or other features suggestive of iron deficiency should therefore prompt appropriate assessment of iron status rather than being labelled simply as a nonspecific inflammatory response [9,10,30].

Medication history should still be reviewed, but anticoagulant use should not ordinarily be regarded as the cause of an isolated high platelet count. Anticoagulants are more relevant to the patient's overall bleeding and thrombotic risk and to management of the underlying condition.

The next question is whether the thrombocytosis is persistent and unexplained. A platelet count that remains elevated after an acute illness or without an obvious reactive explanation warrants review of previous FBCs, clinical history and other blood indices. Persistent unexplained thrombocytosis may require further investigation for a clonal myeloid disorder, particularly when accompanied by other abnormal blood counts, splenomegaly, constitutional symptoms or thrombotic or vasomotor symptoms. Current BSH guidance provides a structured approach to unexplained thrombocytosis, including cases without classical JAK2, CALR or MPL mutations [31].

The absolute platelet count alone does not reliably distinguish reactive thrombocytosis from a clonal disorder. Persistence, pattern and clinical context are more informative than a single elevated result [30,31].

Clinical pearl: A high platelet count is often a marker of what is happening elsewhere in the patient. Before looking for a rare haematological diagnosis, look for the common clinical causes.

Thrombocytosis: Reactive or Persistent?

When a raised platelet count is identified, the assessment should begin by considering the clinical context. Relevant factors include infection, inflammation, recent surgery, tissue injury, blood loss, iron deficiency, malignancy, or previous splenectomy. The trend should then be assessed to determine whether the thrombocytosis is transient or has persisted on previous FBCs. The overall haematological pattern should also be reviewed, including the Hb, red-cell indices, WBC, and other relevant findings. It is important to determine whether there is a convincing reactive cause for the thrombocytosis. If thrombocytosis is persistent or unexplained, particularly when accompanied by other blood-count abnormalities, splenomegaly, constitutional symptoms, or thrombotic or vasomotor symptoms, further investigation and haematology advice should be considered.

Platelets and Thrombosis: When Does the Platelet Abnormality Matter?

A high platelet count can be associated with thrombosis, but thrombocytosis should not automatically be equated with thrombosis risk. The clinical significance depends on whether the thrombocytosis is reactive or clonal and on the patient's wider thrombotic risk profile [30,31].

Reactive thrombocytosis is commonly seen during infection, inflammation, tissue injury and recovery from blood loss or surgery. In these settings, the elevated platelet count is usually a marker of the underlying inflammatory or physiological process rather than an indication for platelet-directed treatment. Management should therefore focus on the underlying cause and on the patient's overall venous and arterial thromboembolism risk [30].

The situation is different in clonal thrombocytosis, particularly essential thrombocythaemia and other myeloproliferative neoplasms. Sustained thrombocytosis in these disorders is associated with both thrombotic and haemorrhagic complications, with risk influenced by factors beyond the platelet count itself [30,31].

The practical question for foundation doctors is therefore not whether a particular platelet number is inherently “dangerous”, but whether the patient has evidence of thrombosis, a clear reactive explanation, or persistent thrombocytosis suggesting an underlying myeloproliferative disorder.

New unilateral leg swelling or pain, unexplained hypoxia or pleuritic chest pain may indicate venous thromboembolism, while focal neurological symptoms, visual disturbance or other acute arterial symptoms require urgent assessment according to the clinical presentation. In a patient with persistent unexplained thrombocytosis, recurrent thrombosis or other features suggestive of a myeloproliferative neoplasm, specialist assessment is appropriate [30,31].

Conversely, a patient with a high platelet count does not necessarily require antiplatelet or anticoagulant treatment simply because the number is elevated. Such treatment depends on the underlying diagnosis and the indication for thromboprophylaxis or antithrombotic therapy.

The same principle applies to patients with very high platelet counts who develop bleeding. In clonal disorders, marked thrombocytosis can be associated with bleeding as well as thrombosis, so the whole haemostatic picture must be considered rather than assuming that a high platelet count protects against bleeding [30].

Beyond the numbers: The question is not “Is the platelet count high enough to cause thrombosis?” but “Why is it high, and does this patient have evidence of clinically important thrombosis or a disorder that increases thrombotic risk?”

The relationship between thrombocytosis, clinical context and thrombotic risk is summarised in Table 8.

**Table 8:** Does thrombocytosis mean thrombosis? Abbreviations: FBC, full blood count; VTE, venous thromboembolism.

Finding	What should it prompt you to think about?
Recent surgery, fracture, infection or inflammation	Reactive thrombocytosis is common; assess the underlying illness and the patient's usual VTE risk
Persistent unexplained thrombocytosis	Review previous FBCs and investigate secondary and clonal causes
High platelets + previous thrombosis	Consider whether an underlying myeloproliferative disorder is contributing
High platelets + new thrombotic symptoms	Assess urgently for the suspected thrombotic event; do not attribute symptoms to the platelet count alone
High platelets + bleeding	Consider the whole haemostatic picture; marked thrombocytosis in clonal disorders can be associated with bleeding as well as thrombosis

Platelet Transfusion and Procedures: When Does the Number Change Management?

Platelet transfusion is an important supportive treatment in selected patients with thrombocytopenia, active bleeding or a planned procedure where the platelet count is considered insufficient for haemostasis. However, the platelet count alone is not an indication for transfusion. The decision should take account of bleeding, the cause and trajectory of thrombocytopenia, the planned procedure, other abnormalities of haemostasis and the patient's overall clinical condition [12,13,32].

For foundation doctors, it is useful to distinguish between therapeutic transfusion, where platelets are given to support a patient who is bleeding, and prophylactic transfusion, where they are given to reduce the risk of bleeding in a patient who is not currently bleeding. The appropriate threshold differs according to the clinical situation.

NICE recommends platelet transfusion for clinically significant bleeding (WHO grade 2) when the platelet count is below 30 × 10⁹/L, with higher thresholds - up to 100 × 10⁹/L - considered for severe bleeding or bleeding at critical sites such as the central nervous system [12].

For non-bleeding adults who are not undergoing an invasive procedure or surgery, NICE recommends prophylactic platelet transfusion below 10 × 10⁹/L, but specifically excludes several conditions in which routine prophylactic transfusion is not recommended, including chronic bone marrow failure, ITP, HIT and thrombotic thrombocytopenic purpura (TTP) [12].

Before an invasive procedure or surgery, NICE recommends considering prophylactic transfusion to raise the platelet count above 50 × 10⁹/L. A higher threshold, for example 50-75 × 10⁹/L, may be appropriate when bleeding risk is particularly high, taking into account the procedure, cause of thrombocytopenia, whether the platelet count is falling, and other abnormalities of haemostasis. For surgery at critical sites such as the central nervous system or posterior segment of the eye, a target above 100 × 10⁹/L may be considered. Conversely, routine prophylactic transfusion is not recommended for low-risk procedures such as central venous cannulation or bone marrow aspiration and trephine biopsy [12].

These thresholds should be regarded as clinical decision points rather than rigid rules. Local Trust or Health Board transfusion policies may provide additional requirements, and specialist haematology, transfusion medicine or procedural advice may be necessary for patients with complex haematological disorders [12,13,32].

Prescribing and Administering Platelets

Once the decision to transfuse has been made, platelet transfusion should follow the local transfusion policy and standard blood-component safety procedures. The indication should be clear and documented, and prescription and blood-component checks should be completed according to local policy [13].

In most circumstances, a single adult dose is given initially rather than automatically prescribing multiple doses. NICE recommends reassessing the patient's clinical condition and checking the platelet count after each transfusion before deciding whether another dose is required [12].

The patient's observations should be monitored before, during and after transfusion according to local policy. Particular attention should be paid to new fever, rigors, rash, hypotension, respiratory symptoms, chest or back pain, or unexpected clinical deterioration. Platelets are associated with recognised transfusion reactions, including febrile and allergic reactions, and bacterial contamination is an important consideration [13,33].

A new fever during or shortly after platelet transfusion should therefore not simply be attributed to the underlying malignancy, infection or chemotherapy. If a transfusion reaction is suspected, stop the transfusion, assess the patient promptly and follow the local transfusion-reaction protocol. If the patient is clinically unstable, an ABCDE assessment and immediate resuscitation take priority. Where bacterial contamination or sepsis is suspected, the patient should be managed through the appropriate sepsis pathway and antibiotics commenced promptly in accordance with local policy and senior or microbiology advice. BSH guidance emphasises immediate recognition and management of potentially life-threatening transfusion reactions and appropriate investigation and reporting [13,33].

For foundation doctors, the practical lesson is therefore not to memorise a transfusion rate or number of units in isolation. The important steps are to confirm the indication, prescribe the appropriate dose, follow local transfusion procedures, monitor the patient, reassess after transfusion, and recognise a possible transfusion reaction early.

Clinical pearl: “Platelets <X” is not a complete transfusion prescription. Always ask: why am I transfusing, what am I trying to achieve, and how will I know whether the patient needs another dose? The platelet count provides a threshold for consideration; the indication, bleeding status, diagnosis, procedure and response to transfusion determine what happens next.

Platelet Red Flags and Escalation

Early senior or haematology input should be considered when significant or ongoing bleeding accompanies thrombocytopenia, when the platelet count is severely abnormal or rapidly falling, or when thrombocytopenia occurs with new abnormalities in other cell lines. Further assessment is warranted when there is a clinically important fall in the platelet count following heparin exposure or another potentially causative drug, or when thrombocytopenia is unexplained or persistent. Similarly, early senior or haematology input should be considered when thrombocytosis is marked, persistent, or unexplained, particularly when there is suspected marrow disease or haematological malignancy.

Senior or specialist input is also appropriate when a platelet abnormality is accompanied by systemic deterioration, or when thrombocytopenia is associated with haemolysis, schistocytes, neurological or renal dysfunction, or abnormal coagulation. Suspected HIT or another thrombotic or haematological emergency should prompt urgent specialist assessment. Finally, further evaluation should be sought when the platelet result does not fit the clinical picture and cannot readily be explained.

Clinical instability or active bleeding requires immediate assessment and escalation, while unexplained or concerning platelet abnormalities in stable patients warrant appropriate investigation and follow-up.

Interpreting the FBC when abnormalities occur together

The FBC is often interpreted by considering Hb, WBC and platelets separately. While this is useful for identifying and characterising individual abnormalities, it can miss an important clinical signal: the relationship between abnormal cell lines. When two or more components of the FBC are abnormal, the combination may provide more information than any single result and should prompt consideration of whether there is a unifying underlying process [1,2,6].

This is an important extension of the Beyond the Numbers approach. The question is no longer simply “Which number is abnormal?” but “What does the overall pattern suggest, and does one process explain the abnormalities across the FBC?” Simultaneous assessment of the red-cell, white-cell and platelet lineages can provide clues to marrow dysfunction, haemolysis, severe infection or haematological disease [1,2,6].

For foundation doctors and other early-career clinicians, this does not require making a specialist haematological diagnosis from the FBC alone. Instead, it requires recognising when an apparently isolated abnormality is part of a wider pattern, identifying potentially important causes, and knowing when further investigation or senior review is required.

A useful starting question is: “Is there one abnormality, or are several cell lines telling me the same story?”

Multiline Abnormalities: Recognising the Wider Pattern

When more than one cell line is affected, the first step is to establish whether the abnormalities are genuine and whether they are new. Previous FBC results are particularly valuable because they distinguish a long-standing pattern from an acute change. A stable, mildly abnormal FBC may require a different approach from a previously normal FBC that has developed new anaemia, neutropenia and thrombocytopenia over several days [1,2].

Four broad mechanisms provide a useful starting point when evaluating abnormalities involving more than one blood cell lineage. Reduced production occurs when the bone marrow is unable to adequately produce one or more cell lines, as may occur with marrow failure, marrow infiltration, nutritional deficiency, chemotherapy, or other drug effects. Increased destruction or consumption refers to processes in which circulating cells are lost through mechanisms such as haemolysis, immune destruction, DIC, or TMA.

A third mechanism is redistribution or sequestration, whereby cells are removed from the circulating pool, for example through hypersplenism. Finally, reactive or systemic processes may produce abnormalities across more than one lineage in response to infection, inflammation, bleeding, physiological stress, or other systemic illness [1,6].

These mechanisms are not mutually exclusive. A patient may have more than one process operating simultaneously; for example, an acutely unwell patient may have pre-existing iron deficiency together with inflammation, bleeding or medication-related marrow suppression. The aim is therefore not to force every abnormality into a single diagnosis, but to ask whether the pattern is coherent and clinically plausible.

Bicytopenia

When two cell lines are abnormal, the clinician should broaden the assessment rather than interpreting each abnormality independently. Bicytopenia is a pattern, not a diagnosis, and its significance depends on which cell lines are affected, whether the abnormalities are new or progressive, and the clinical context.

Some combinations provide useful clinical prompts, as shown below. 

Anaemia + thrombocytopenia: consider blood loss, haemolysis, nutritional deficiency, medication effects, infection, liver or splenic disease, and bone marrow pathology. Anaemia with thrombocytopenia accompanied by schistocytes or evidence of haemolysis should prompt urgent consideration of TMA or another consumptive process [6,27].

Anaemia + neutropenia: consider nutritional deficiency, medication or chemotherapy effects, infection, systemic disease and impaired marrow production. The clinical context and trend are particularly important [1,16,17].

Thrombocytopenia + neutropenia: consider acute infection or sepsis, drug or chemotherapy effects, nutritional deficiency and bone marrow disorders. In a clinically unwell patient, this combination should not be dismissed as an incidental laboratory finding [1,4,5,17].

A blood film and targeted investigations may be appropriate when the cause is unclear, particularly where there is a rapid change, abnormal morphology or concern about marrow disease [1,6].

Pancytopenia

Pancytopenia refers to a reduction in all three major circulating blood cell lines: red cells, white cells and platelets. It may reflect a reversible systemic or nutritional problem, but it can also be a manifestation of significant bone marrow failure, marrow infiltration or haematological malignancy. The finding therefore warrants structured assessment rather than being approached as three separate abnormalities [6,34].

The first step is to confirm that pancytopenia is genuine and assess the patient, particularly for bleeding, fever, infection, haemodynamic instability or other evidence of acute deterioration. Previous FBC results should then be reviewed to establish whether the cytopenias are longstanding or represent a new or rapidly evolving process.

Initial assessment commonly includes a reticulocyte count, which helps determine whether the bone marrow is responding appropriately to anaemia. A low or inappropriately normal reticulocyte response suggests impaired red-cell production, whereas an increased response may support blood loss or haemolysis. A blood film should also be reviewed, as it may reveal blasts, abnormal precursor cells, dysplastic changes, schistocytes, or a leucoerythroblastic picture that is not apparent from the automated FBC. Concerning findings, particularly circulating blasts or features suggestive of acute leukaemia, should prompt urgent senior and haematology discussion rather than routine follow-up.

Targeted investigations should be guided by the clinical history and examination and may include haematinics, haemolysis markers, liver and renal function, inflammatory or infective investigations, medication and chemotherapy review, and assessment for liver disease or splenomegaly. Persistent, unexplained, or progressive pancytopenia should also raise the possibility of impaired or infiltrated bone marrow, including aplastic anaemia, myelodysplastic syndromes, acute leukaemia, and other marrow-infiltrative processes [6,34].

Pancytopenia alone does not establish a diagnosis of malignancy. The possibility becomes more important when it is accompanied by abnormal blood-film findings, constitutional symptoms, lymphadenopathy, splenomegaly or progressive deterioration [6,34].

The practical aim is therefore not to determine the exact cause of pancytopenia from the initial FBC. It is to recognise the pattern, identify immediate risks, initiate appropriate first-line investigations and ensure that potentially serious causes are escalated promptly.

Clinical pearl: Pancytopenia is a pattern, not a diagnosis. Confirm it, assess the patient, check reticulocytes and the blood film, look for reversible causes, and escalate early when the pattern or clinical picture is concerning.

Recognising Important Cross-Line Patterns

One of the main advantages of interpreting the FBC as a whole is the ability to recognise relationships between cell lines. For foundation doctors, these patterns should be used as clinical prompts rather than diagnostic equations. The presence of a particular combination does not establish a diagnosis; instead, it should prompt the clinician to ask whether the pattern is consistent with the patient's clinical state, whether it is new or persistent, and whether further investigation is required.

Important cross-line FBC patterns and the questions they should prompt are summarised in Table 9.

**Table 9:** Cross-line FBC patterns: what should they prompt you to consider? Abbreviations: DIC, disseminated intravascular coagulation; FBC, full blood count; LDH, lactate dehydrogenase; TMA, thrombotic microangiopathy.

FBC pattern	What to consider	What should prompt further thought?
Microcytic anaemia + thrombocytosis	Iron deficiency with reactive thrombocytosis; inflammation or another reactive process	Evidence of iron deficiency or blood loss; persistent or unexplained thrombocytosis
Anaemia + thrombocytopenia + evidence of haemolysis	TMA, DIC or another destructive/consumptive process	Schistocytes, raised LDH, low haptoglobin, jaundice, renal or neurological abnormalities, abnormal coagulation or clinical deterioration
Anaemia + neutropenia	Nutritional deficiency, medication/chemotherapy effect, infection, systemic disease or impaired marrow production	New or progressive abnormality, recent drug exposure, infection, nutritional risk or another cytopenia
Pancytopenia	Marrow/systemic process, nutritional deficiency, infection, drug or chemotherapy effect, hypersplenism	Confirmation of all three lineages, reticulocyte response, blood film, clinical deterioration or lack of a reversible explanation
Neutropenia + thrombocytopenia with acute deterioration	Severe infection/sepsis, drug effect, consumption or marrow pathology	Fever, hypotension, organ dysfunction, recent medication/chemotherapy or rapidly falling counts

These patterns should not be interpreted in isolation. For example, microcytic anaemia with thrombocytosis may occur in iron deficiency, but the same pattern in a patient with an acute inflammatory illness may reflect a reactive response. Similarly, anaemia with thrombocytopenia is not synonymous with TMA: the presence of haemolysis, schistocytes, organ dysfunction or other compatible clinical features is what should increase concern [6,27].

The combination of anaemia, thrombocytopenia and haemolysis is particularly important because it can represent a time-critical process. TMA and DIC should be considered when thrombocytopenia occurs alongside evidence of red-cell destruction, particularly when the patient is systemically unwell. A blood film demonstrating schistocytes can support the assessment, but morphology should be interpreted alongside haemolysis markers, coagulation results and the clinical picture rather than used as a stand-alone diagnostic test [6,27,29].

Likewise, a combination of neutropenia and thrombocytopenia should not automatically be labelled as marrow failure. Infection, medication effects and acute systemic illness are important considerations. However, when abnormalities involve several lineages, are progressively worsening, or remain unexplained, the threshold for reviewing the blood film and seeking senior or haematology advice should be lower [1,16,17].

The trend is often what converts a pattern from a potentially minor finding into a clinically important one. A patient with long-standing mild anaemia and stable platelets may require routine investigation, whereas a previously normal FBC that develops rapidly progressive anaemia, neutropenia and thrombocytopenia requires a different level of urgency. Comparison with previous results is therefore an important component of integrated FBC interpretation [1,2,6].

The purpose of pattern recognition is not to diagnose from the FBC alone. It is to identify when the combination of results changes the level or direction of clinical investigation.

Clinical pearl: Patterns are prompts, not diagnoses. Use the combination of abnormal cell lines to decide what question to ask next - not to decide the diagnosis from the FBC alone.

When the FBC Does Not Fit the Patient

The converse of pattern recognition is equally important: sometimes the FBC does not fit the clinical picture.

An unexpected laboratory result can be clinically misleading if it is accepted without verification. A markedly abnormal result in a patient who appears well, a sudden unexplained change from previous results, or a result that conflicts with other investigations should prompt reassessment rather than an immediate assumption that the patient has developed a new disease [1-3].

The key principle is: If the laboratory result does not fit the patient, stop and reassess rather than forcing the patient to fit the number.

Unexpected abnormalities should first be considered in relation to the rest of the FBC and differential, previous results and any analyser flags or sample issues. Automated analysers are highly useful but do not eliminate pre-analytical or analytical problems. Platelet clumping can produce pseudothrombocytopenia, while other artefacts can affect red-cell indices or cell counts. Unusual histograms, analyser flags or a result that is markedly discordant with the clinical picture should therefore prompt consideration of a spurious result [1,3,25].

When the patient is clinically stable and the result is unexpected or inconsistent with previous testing, repeating the FBC may establish whether the abnormality is persistent and genuine. However, not every unexpected result requires immediate repetition, and an abnormal result should not simply be “rechecked” when the patient is clearly unwell and urgent clinical assessment is required.

This distinction is important: clinical urgency determines whether verification comes before action or occurs in parallel with it. In a deteriorating patient, clinical assessment and treatment should not be delayed simply because the laboratory result has not yet been confirmed.

Previous FBCs may reveal that an abnormality is longstanding, improving, worsening or genuinely new. A modest abnormality that has remained stable for years may carry a different significance from the same value appearing abruptly over 24-48 hours. Conversely, a patient with progressively changing counts may require escalation even if individual values have not yet crossed a conventional threshold of concern [1,2].

If the result remains unexplained, laboratory discussion and peripheral blood-film review may be appropriate, particularly when there are analyser flags, unexplained cytopenias, suspected haemolysis, abnormal cell populations or a discrepancy between the automated count and the clinical presentation. A blood film may identify platelet clumping, blasts, abnormal precursor cells, schistocytes or other morphological features that substantially alter the differential diagnosis [1,3,6].

The clinician should then return to the patient: Is the patient actually bleeding? Is there fever or evidence of infection? Are they haemodynamically stable? Is there evidence of haemolysis or organ dysfunction? Have there been recent medications, chemotherapy, surgery or significant illness? Are there previous blood results that explain the current pattern? Does the rest of the clinical assessment support the laboratory result?

Certain situations should lower the threshold for senior review or further investigation, including: a rapid or unexpected change from previous FBC results; several cell lines becoming abnormal without an obvious explanation; blasts or other abnormal cells on the blood film; anaemia with thrombocytopenia and evidence of haemolysis; severe neutropenia with fever or systemic deterioration; significant bleeding associated with thrombocytopenia; persistent unexplained cytopenias or other abnormalities; or a markedly abnormal laboratory result that is inconsistent with the clinical presentation.

The response should remain proportionate to the clinical situation. A discordant result in a stable patient may allow time for confirmation, repeat testing and review of the sample. In a deteriorating patient, however, clinical assessment and treatment should not be delayed simply because the laboratory result has not yet been confirmed.

Discordant FBC results should therefore be approached using the original Beyond the Numbers framework, with clinical urgency determining whether verification precedes action or occurs alongside it.

Clinical pearl: When the FBC and the patient disagree, don't automatically trust either one. Reassess the patient, verify the result and look at the trend before deciding what the number means.

When to Escalate: Recognising When the FBC Is Bigger Than an Isolated Abnormality

Most FBC abnormalities encountered by foundation doctors will not require immediate specialist input. However, when abnormalities involve several cell lines, change rapidly, or occur alongside clinical deterioration, the FBC may be signalling a more significant underlying process.

The practical question is: “When should I stop managing this as an isolated laboratory abnormality?”

Escalation does not require a definitive haematological diagnosis. It requires recognition that the pattern, trend or clinical condition is concerning enough that further assessment should not be delayed.

FBC patterns that should prompt consideration of escalation are summarised in Table 10.

**Table 10:** FBC patterns that should prompt escalation Abbreviations: FBC, full blood count; TMA, thrombotic microangiopathy; DIC, disseminated intravascular coagulation.

Finding	Why it matters	Practical action
Multiple significantly abnormal cell lines	May indicate systemic illness, marrow suppression/failure, nutritional deficiency, infection or haematological disease	Assess the patient, review the trend and consider senior/haematology discussion
Rapidly changing blood counts	A falling or rapidly evolving pattern may be more clinically significant than a single abnormal value	Review previous FBCs and investigate the cause promptly
Blasts or concerning blood-film findings	May indicate acute leukaemia, marrow disease or another serious haematological process	Urgent senior and haematology discussion; do not rely on routine follow-up
Pancytopenia with systemic illness	May reflect severe infection, drug/chemotherapy effect, marrow failure or other significant systemic pathology	Assess urgently and initiate appropriate investigations; escalate according to clinical condition
Thrombocytopenia + evidence of haemolysis	Raises concern for TMA, DIC or another consumptive/destructive process	Urgent clinical assessment and senior/haematology involvement
Severe neutropenia + fever or suspected sepsis	Risk of neutropenic sepsis and rapid clinical deterioration	Treat as a clinical emergency according to local neutropenic-sepsis/sepsis guidance [4,5,17]
Significant bleeding with an abnormal platelet count	The clinical consequence of thrombocytopenia may be more important than the number alone	Immediate clinical assessment, appropriate haemorrhage management and senior/haematology input
Persistent unexplained cytopenias or suspected marrow failure/malignancy	Persistent abnormalities across cell lines require consideration of an underlying marrow or systemic disorder	Review previous results, blood film and initial investigations; seek senior/haematology advice

These findings are triggers for escalation, not diagnoses. The appropriate response depends on the severity of the abnormality, the rate of change, the patient's clinical condition and the suspected underlying cause. A stable patient with a longstanding abnormality may require planned investigation, whereas a similar laboratory pattern in a deteriorating patient may require immediate assessment.

The same principle applies to blood-film findings. Circulating blasts or other features concerning for acute leukaemia should not be managed as an incidental abnormal FBC finding. Similarly, thrombocytopenia accompanied by haemolysis, schistocytes or new renal or neurological dysfunction should prompt urgent consideration of a TMA or other serious consumptive process [6,27].

Before deciding that an abnormal FBC can simply be followed up, ask: Is the patient unwell? Are multiple cell lines affected? Are the counts changing rapidly? Is there bleeding, haemolysis, fever or organ dysfunction? Is the blood film concerning? Is there a plausible explanation-or is the abnormality unexplained? If one or more of these questions raises concern, stop treating the FBC as an isolated laboratory abnormality and escalate appropriately.

For foundation doctors, escalation does not mean that a definitive haematological diagnosis must already have been established. It means recognising that the pattern is potentially significant and that further assessment should not be delayed.

Clinical pearl: You do not need to know the diagnosis before escalating. Recognising that the FBC pattern is concerning is itself an important clinical skill.

Discussion

Principal Contribution of the Beyond the Numbers Framework

The principal contribution of this review is to frame FBC interpretation as a process of clinical reasoning rather than numerical recognition. Reference ranges remain an essential starting point, but they do not determine clinical significance in isolation. The proposed Beyond the Numbers framework instead emphasises the relationship between the laboratory result, the patient's clinical condition, previous results, the pattern across cell lines, and the appropriate clinical response [1,2,6].

A key feature of the framework is the distinction between clinical urgency and laboratory verification. This distinction is important because the same numerical abnormality may have very different implications depending on the patient's physiological state and the speed with which the abnormality has developed [1,4-6,17].

The framework also places greater emphasis on relationships between abnormalities rather than treating each cell line independently. Trends and cross-line patterns can provide clinically important information that may be missed when results are considered in isolation. Anaemia with thrombocytopenia, neutropenia with thrombocytopenia, or pancytopenia, for example, may prompt consideration of systemic, nutritional, drug-related, consumptive or marrow processes [1,6,34]. Such combinations should not be treated as diagnostic equations; rather, they identify questions that require further clinical assessment and investigation. In this respect, the framework is intended to support recognition of potentially important patterns without encouraging premature diagnostic closure.

An additional practical contribution is its explicit link between interpretation and escalation. Clinicians do not always need to establish the underlying diagnosis before deciding that a patient requires urgent review. Concerning blood-film findings, rapidly evolving or multiple cytopenias, thrombocytopenia with haemolysis, severe neutropenia with fever or suspected sepsis, significant bleeding, and persistent unexplained abnormalities are examples in which recognising the pattern may be sufficient to trigger appropriate escalation [4,5,11,17,34]. The framework therefore aims to support proportionate action under conditions of clinical uncertainty rather than requiring diagnostic certainty before action is taken.

Relevance to Foundation Doctors and Early-Career Clinicians

The framework is particularly relevant to foundation doctors because FBC abnormalities are encountered frequently and often before a definitive diagnosis has been established. Early-career clinicians may recognise that a result is outside the reference range without yet having developed the pattern-recognition and prioritisation skills required to determine its significance. A structured interpretive approach can help bridge this gap by providing a consistent way of moving from an abnormal result to an assessment of urgency and an appropriate next step [1,2].

Importantly, the framework is not intended to turn foundation doctors into specialist haematologists or to encourage diagnosis from the FBC alone. Its educational value lies in supporting an appropriate level of clinical reasoning: recognising whether an abnormality is new or longstanding, identifying whether other cell lines are involved, considering whether the finding fits the clinical picture, recognising potentially dangerous patterns, and knowing when senior or specialist input is required. This may be particularly useful in environments where clinicians must interpret laboratory results alongside competing clinical priorities and incomplete diagnostic information.

The approach is also transferable across clinical settings because it is not dependent on a single disease or cell-line abnormality. The same reasoning can be applied to anaemia, white-cell abnormalities, thrombocytopenia, thrombocytosis and combinations of abnormalities, while condition-specific guidance remains responsible for defining subsequent investigation and treatment [4,5,11,12,17,28,31]. In this sense, Beyond the Numbers functions as a cognitive scaffold for clinical judgement rather than as a diagnostic algorithm.

Relationship to Existing Guidance and Clinical Pathways

The framework should be understood as complementary to, rather than a replacement for, established clinical guidance. National and specialty-specific guidance remains essential for decisions involving defined diagnostic pathways, treatment thresholds, transfusion, neutropenic sepsis, suspected haematological malignancy, persistent cytopenias and other high-risk clinical scenarios [4,5,11,12,28,31]. Similarly, local protocols may determine the practical thresholds and escalation routes used within individual organisations.

The proposed framework occupies an earlier stage of this process. Its purpose is to help clinicians recognise when a result requires further attention and when a condition-specific pathway should be considered. It therefore addresses the interpretive step between receiving an FBC result and selecting the most appropriate clinical pathway. This positioning is particularly relevant where an abnormality is not sufficiently specific to identify a diagnosis but is sufficiently concerning to require further assessment.

The framework may also help reduce inappropriate reliance on isolated numerical thresholds. Thresholds remain clinically important, but their meaning depends on the patient, trajectory, associated abnormalities and clinical circumstances [5,12,28,31]. Conversely, a result that is not dramatically abnormal should not necessarily be regarded as benign when accompanied by clinical deterioration or a concerning pattern [4,6,17]. The framework therefore provides a context in which established thresholds and guidelines can be applied more appropriately, rather than attempting to replace them.

Educational Implications and Future Evaluation

The educational value of Beyond the Numbers lies in its potential to make an otherwise fragmented interpretive task more explicit and teachable. The framework could be incorporated into undergraduate and postgraduate teaching, ward-based supervision, clinical induction and visual decision-support materials. Its emphasis on urgency, verification, trend, pattern, context and action may also provide a common language for discussing FBC interpretation between trainees and senior clinicians.

However, the framework is currently a conceptual and educational model rather than a prospectively validated clinical intervention. Its ability to improve diagnostic accuracy, recognition of deterioration, appropriateness of investigation, escalation decisions or clinician confidence has not been established. Future evaluation should therefore assess whether structured use of the framework improves interpretation of clinically relevant FBC patterns and whether it influences decision-making without increasing unnecessary investigation or referral. Studies involving foundation doctors and other early-career clinicians could examine usability, cognitive load, decision accuracy and the appropriateness and timeliness of escalation in realistic clinical scenarios.

Overall, Beyond the Numbers offers a practical interpretive scaffold between laboratory data and clinical decision-making. Its central contribution is not another catalogue of haematological diagnoses, but a structured way of deciding when an FBC abnormality matters, what it may represent in context, and what should happen next. Its appropriate role is therefore as an adjunct to clinical judgement and established disease-specific guidance, particularly for clinicians developing the practical reasoning skills required to interpret FBC results safely and proportionately.

Limitations

This review has several limitations that should be considered when interpreting the proposed Beyond the Numbers framework. First, this is an educational narrative review rather than a systematic review or formal clinical practice guideline. The literature was selected to support a practical approach to common FBC abnormalities encountered by foundation doctors and early-career clinicians, rather than to provide an exhaustive synthesis of all available evidence for each haematological abnormality. Consequently, the framework should be viewed as an educational aid to clinical reasoning rather than as a validated diagnostic or decision-making tool.

Second, the clinical significance of individual FBC abnormalities and the thresholds at which investigation, treatment or escalation is recommended may vary according to national guidance, specialty-specific recommendations and local hospital or health-board protocols. This is particularly relevant to areas such as platelet transfusion, management of neutropenic sepsis, anticoagulation in thrombocytopenia and referral pathways for suspected haematological malignancy [5,11,12,28,31]. Clinicians should therefore use current local and national guidance when making management decisions. The framework is intended to help clinicians recognise when such guidance may need to be consulted or activated, rather than to replace it.

Third, the FBC has important diagnostic limitations. Although patterns within the FBC can provide valuable clinical clues, the blood count rarely establishes a diagnosis independently. Similar numerical abnormalities may occur in very different clinical conditions, while clinically important disease may occasionally occur despite apparently modest or non-specific changes. Interpretation therefore requires integration with the patient's history, examination, previous results, medications, other investigations and overall clinical trajectory [1,2,6].

The framework has also not yet been prospectively evaluated to determine whether its use improves diagnostic accuracy, recognition of deterioration, investigation patterns, escalation decisions or patient outcomes among foundation doctors. Its educational value and usability therefore remain to be formally assessed. Future work could evaluate the framework in undergraduate or postgraduate education and determine whether structured FBC interpretation improves confidence, pattern recognition and appropriate escalation.

Finally, the framework intentionally prioritises common and clinically important situations encountered in early clinical practice rather than providing comprehensive coverage of specialist haematology. Rare disorders and complex diagnostic pathways are therefore beyond its intended scope. Where an FBC abnormality is severe, rapidly changing, unexplained, associated with significant symptoms, or inconsistent with the clinical picture, appropriate senior or specialist advice remains essential.

These limitations reinforce the intended role of Beyond the Numbers: it is a structured educational approach designed to support clinical reasoning, not a substitute for clinical judgement, specialist expertise or disease-specific guidance.

## Conclusions

The FBC is one of the most frequently encountered investigations in clinical practice, but its interpretation requires more than identifying values outside a laboratory reference range. Its clinical meaning depends on the condition of the patient, the validity and trajectory of the result, the pattern across the blood count, and the wider clinical context.

The Beyond the Numbers framework provides a practical approach to this clinical reasoning. It is intended to help foundation doctors and other early-career clinicians distinguish situations requiring immediate action from those that can be approached systematically, while recognising when further investigation, senior review or specialist input is required. The framework complements rather than replaces clinical judgement and condition-specific guidance.

Its central message is to prioritise clinical urgency and use a structured approach to determine the significance of an abnormal FBC and the appropriate next action.
